# Monitoring Epithelial–Mesenchymal Transition of Pancreatic Cancer Cells via Investigation of Mitochondrial Dysfunction

**DOI:** 10.3390/mps3020032

**Published:** 2020-04-27

**Authors:** Jae Jun Sim, Keun-Yeong Jeong

**Affiliations:** MetiMedi Pharmaceuticals Co., Research Center, Incheon 22006, Korea; genesis0804@hanmail.net

**Keywords:** pancreatic cancer cell, hypoxia, ROS, mitochondria, membrane potential, epithelial–mesenchymal transition

## Abstract

In this protocol, we introduced a method of measuring mitochondrial dysfunction to confirm the epithelial–mesenchymal transition (EMT) in pancreatic cancer cells under a hypoxic environment. There are many expertized and complicated methods to verify EMT. However, our methods have indicated that EMT can be identified by examining changes in reactive oxygen species (ROS) generation and membrane potential in mitochondria. To demonstrate whether the changes in the indicators of mitochondrial dysfunction are correlative to EMT, cell morphology, and expression of E-cadherin and N-cadherin were additionally observed. The results verified that a decrease in membrane potential and an increase in ROS in mitochondria were associated with EMT of pancreatic cancer cells. This protocol would be useful as a basis for providing an additional indicator for changes in the tumor microenvironment of pancreatic cancer cells relating to EMT under a hypoxic environment.

## 1. Introduction

The hypoxic environment induced by central region necrosis in the tumor burden can cause various changes in the molecular mechanisms of cancer and lead to an advanced stage of cancer such as metastasis [1,2]. In particular, cancer cells alter the function of the mitochondria to adapt hypoxia including membrane potential and oxidative phosphorylation so that it can perform optimal metabolism for energy supply [3]. Mitochondria are the dynamic organelles that can involve regulating cell energy metabolism and produce the majority of adenosine triphosphate, which is needed to maintain cell viability and cellular physiology [4]. Cancer cells retain impaired mitochondrial membrane potential when incubated under oxygen-limiting conditions [5]. The stable membrane potential should be maintained to avoid uncontrolled reactive oxygen species (ROS) production. However, a lack of oxygen induces reductive carboxylation in mitochondria is the leading cause of reactive oxygen species (ROS) generation in cancer cells [3]. This feature is a driving force that maintains viability by having tumor hypoxia tolerance [6]. The leading feature of pancreatic cancer is that it is desmoplastic, which refers to the presence of highly mesenchymal-like substrates, and is thus involved in pancreatic cancer growth, metastasis to distant organs, and drug resistance [7,8]. Therefore, various investigations on pancreatic cancer are being performed to confirm a mesenchymal feature, as it would be an optimal approach that reflects the actual tendency of pancreatic cancer [9]. There is a variety of epithelial–mesenchymal transition (EMT) markers including transcription modulators and signaling molecules, such as fibronectin, vimentin, SNAI1, Twist, cadherin protein family, and so on [9]. However, few reports have described the testifying EMT induction by changes in mitochondria dysfunction in pancreatic cancer cells. Our ultimate goal of this study is to introduce a method of measuring mitochondrial membrane potential or ROS generation to confirm the EMT in pancreatic cancer cells under hypoxia. To verify whether the changes in the indicators of mitochondrial dysfunction are correlative to EMT, cell morphology, and expression of E-cadherin and N-cadherin were additionally observed.

## 2. Experimental Design

### 2.1. Materials

- AsPC-1 (American Type Culture Collection, Manassas, VA, USA; Cat. no.: CRL-1682).
- RPMI1640 (Welgene, Daegu, Korea; Cat. no.: LM 011-02).
- Fetal bovine serum (Welgene, Daegu, Korea; Cat. no.: S 101-01).
- Penicillin-Streptomycin solution (Welgene, Daegu, Korea; Cat. no.: LS 202-02).
- Phosphate buffered saline (Welgene, Daegu, Korea; Cat. no.: LB 001-01).
- Trypsin-EDTA solution (Merck, Darmstadt, Hessen, Germany; Cat. no.: 59418C).
- 100 mm culture dishes (Sigma, St. Louis, MO, USA; CLS430167).
- 15 mL tubes (Sigma, St. Louis, MO, USA; CLS430791).
- 6-well plate (Sigma, St. Louis, MO, USA; SIAL0506).
- Mitochondrial superoxide indicator (Invitrogen, Carlsbad, CA, USA; M36008).
- Mitochondria tracking fluorogenic dye (Invitrogen, Carlsbad, CA, USA; M7514).
- bisBenzimide H 33342 trihydrochloride (Invitrogen, Carlsbad, CA, USA; H3570).
- Tetramethylrhodamine, methyl ester (Invitrogen, Carlsbad, CA, USA; T668).
- 1X Hanks’ Balanced Salt solution (Merck, Darmstadt, Hessen, Germany; Cat. no.: H1641).
- Paraformaldehyde (Biosesang, Seongnam, Korea; Cat. no.: PC2031-050-00).
- 5 mL flow cytometry and fluorescence-activated cell sorting tubes (Corning Life Sciences, Oneonta, NY, USA; Cat. no.: 38055).
- Cell imaging dishes (Effendorf, Hamburg, Germany; Cat. no.: 30780009).
- E-cadherin antibody (Invitrogen, Carlsbad, CA, USA; PA5-32178).
- N-cadherin antibody (Invitrogen, Carlsbad, CA, USA; PA5-85916).
- Bio-coated coverslip (BD Bioscience, San Jose, CA, USA; Cat. no.: 354085).
- streptavidin fluorescein-conjugated anti-rabbit secondary antibody (Santa Cruz Biotechnology, CA, USA; Cat. no.: sc-2359).
- Slide glass (Corning Life Sciences, Oneonta, NY, USA; Cat. no.: 2947-75X25).
- Mounting medium with DAPI (Vector Laboratories, Burlingame, CA, USA; Cat. no.: H-1500).
- 20 μL Pipette (Effendorf, Hamburg, Germany; Cat. no.: 3121000031).
- 200 μL Pipette (Effendorf, Hamburg, Germany; Cat. no.: 3121000082).
- 1000 μL Pipette (Effendorf, Hamburg, Germany; Cat. no.: 3121000120).

### 2.2. Equipment

- Hypoxia chamber (COY Laboratory, Grass Lake Charter Township, MI, USA; Cat. no.: 05150112019).
- 37 °C incubator (Benchmark Scientific, Edison, NJ, USA; Cat. no.: H2200-h).
- Inverted microscope (Nikon, Tokyo, Japan; Cat. no.: Ts2).
- Optical microscope (Leica, Wetzlar, Germany; Cat. no.: DM1000 LED).
- Centrifuge (Labogene, Seoul, Korea; Cat. no.: 1580MGR).
- CO_2_ Incubator (Thermo Fisher Scientific, Waltham, MA, USA; Cat. no.: 13-998-086PM).
- Flow cytometry and fluorescence-activated cell sorting machine (BD bioscience, Franklin Lakes, NJ, USA; Cat. no.: 342975).
- Confocal microscope (Nikon, Tokyo, Japan; Cat. no.: A1+).

## 3. Procedure

### 3.1. Cell Culture and Subculture


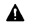
 The pancreatic cancer cells (AsPC-1) were cultured under two condition: a humidified atmosphere at 37 °C containing 19% oxygen, 5% carbon dioxide, and 76% nitrogen (normoxia) and hypoxia maintained a humidified atmosphere at 37 °C containing 1% oxygen, 5% carbon dioxide, and 94% nitrogen. The hypoxia chamber is designed to possible to handle materials inside.

1. AsPC-1 cells were grown in RPMI1640 media supplemented with 10% fetal bovine serum, 100 IU/mL penicillin, and 100 μg/mL streptomycin.
2. Seven days later, wash the cell with 1× phosphate-buffered saline (PBS) without Ca^2+^/Mg^2+^. Then, shake the plate gently.
3. Pipette 1 mL trypsin-EDTA onto the washed cell monolayer. Gently rock the dishes so that the trypsin completely cover their surfaces.
4. Incubate the culture dish at 37 °C for 5 min.
5. Resuspend the cells in fresh serum-containing medium to inactivate the trypsin.
6. Transfer the cell suspension to a 15 mL tube and centrifuge at 112× *g* for 3 min, and then remove the supernatant.
7. Resuspend each cell pellet in 3 mL of pre-warmed culture medium.
8. Dilute cell suspension to 1 × 10^5^ per 100 mm culture dish containing pre-warmed culture medium.
9. Incubate the culture dishes in a humidified atmosphere at 37 °C containing 5% carbon dioxide before each experiment.

### 3.2. Flow Cytometry and Fluorescence-Activated Cell Sorting Analysis for Measuring Mitochondrial ROS


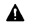
 Pancreatic cancer cells were gated according to physical parameters and aggregates were removed from the analysis. All cells were included in the analysis without consideration of cell viability. The content of mitochondrial ROS was then measured in each condition (unstained, normoxia, hypoxia, and H_2_O_2_) by mitochondrial superoxide indicator.

1. Culture 1 × 10^5^ and 3 × 10^5^ AsPC-1 cells in a 6-well plate (2 mL culture medium per well) and incubate under the normoxia or hypoxia for 48 h, respectively. And then remove the culture medium using suction.
2. Wash the cell with 1 mL PBS per well.
3. Add 5 μM fluorescent mitochondrial superoxide indicator in a serum-free growth medium at 37 °C in the dark for 10 min.
4. Wash the cell with 1 mL PBS per well.
5. Add 500 μL trypsin for 3 min at 37 °C in the dark.
6. Resuspend the cells in 2 mL fresh serum-containing medium, transfer the cell suspension to a 15 mL tube, and centrifuge at 112× *g* for 3 min.
7. Wash the collected cells twice with 10 mL PBS and then centrifuge at 112× *g* for 3 min.
8. Remove supernatant and add 300 μL PBS.
9. Transfer the cells to fluorescence-activated cell sorting tubes (5 mL round-bottom polystyrene tubes).
10. Analyze the mitochondria ROS signal using the flow cytometry and fluorescence-activated cell sorting machine at the excitation wavelength of 582 nm (FL-2).

### 3.3. Confocal Imaging for Measuring Mitochondria ROS

1. Culture 1 × 10^5^ and 3 × 10^5^ AsPC-1 cells in a cell imaging dish (2 mL culture medium per well) and incubate under the normoxia or hypoxia for 48 h, respectively.
2. Wash the cells using 1 mL PBS per well.
3. Remove the PBS from the cell imaging dish.
4. Add 5 μM fluorescent mitochondrial superoxide indicator in serum-free growth medium at 37 °C in the dark for 10 min.
5. Add 200 nM fluorescent mitochondria tracker and nuclear marker (bisBenzimide H 33342 trihydrochloride) for 15 min at 37 °C in the dark.
6. Wash the cell with 1 mL 1× Hank’s Balanced Salt Solution (HBSS) per well and then shake the plate gently.
7. Capture cell images consecutively for ROS fluorescent signal (emission range: 570–620 nm), mitochondria tracker (emission range: 500–550 nm), and bisBenzimide H 33342 trihydrochloride (Hoechst 33342; emission range: 425–475 nm) using the laser scanning confocal microscope.

### 3.4. Confocal Imaging for Measuring Mitochondrial Membrane Potential

1. Culture 1 × 10^5^ and 3 × 10^5^ AsPC-1 cells on a cell imaging dish (2 mL culture medium per dish) and incubate under the normoxia or hypoxia for 48 h, respectively.
2. Wash the cell using 1 mL PBS per well and then shake the plate gently.
3. Remove the PBS from the cell imaging dish.
4. Add 200 nM tetramethylrhodamine, methyl ester (TMRM) in serum-free growth medium at 37 °C in the dark for 30 min.
5. Wash the cell using 1 mL PBS per well and then shake the plate gently.
6. Add 200 nM fluorescent mitochondria tracker and Hoechst 33342 for 15 min at 37 ℃ in the dark.
7. Wash the cell with 1 mL 1× HBSS per dish and then shake the plate gently.
8. Capture cell fluorescence consecutively for TMRM (emission range: 570–620 nm), mitochondria tracker (emission range: 500–550 nm), and Hoechst 33342 (emission range: 425–475 nm) using the laser scanning confocal microscope.

### 3.5. Cell Morphology and EMT-Like Cell Counting

1. Culture 1 × 10^6^ and 3 × 10^6^ AsPC-1 cells in a 6-well plate and incubate under the normoxia or hypoxia for 72 h, respectively.
2. After 72 h, add 2 mL 4% paraformaldehyde solution for cell fixation.
3. After 20 min, take out the culture dishes from the normoxia and hypoxia incubator.
4. Wash the cells three times with 3 mL PBS and then remove the PBS using suction.
5. Add 3 mL fresh PBS to avoid drying.
6. Capture cell morphology using an inverted microscope.
7. Count the number of EMT-like cells using the optical microscope.

### 3.6. Confocal Analysis for Measuring E-Cadherin and N-Cadherin


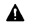
 It is recommended to conduct experiments by blocking the light at the stage containing the fluorescent material.

1. Culture 1 × 10^5^ and 3 × 10^5^ AsPC-1 cells on a bio-coated coverslip in a 6-well plate (2 mL culture medium per dish) and incubate under the normoxia or hypoxia for 48 h, respectively.
2. After 48 h, add 2 mL 4% paraformaldehyde solution for cell fixation.
3. After 20 min, take out the culture dishes from the normoxia and hypoxia incubator.
4. Wash the cells three times with 3 mL PBS and then remove the PBS using suction.
5. Incubate for 15 h at 4 °C with the E-Cadherin or N-Cadherin primary antibody (1:300 dilution using PBS).
6. Wash the cells three times with 3 mL PBS and then remove the PBS using suction.
7. Incubate for 15 h at 4 °C with the streptavidin fluorescein-conjugated anti-rabbit secondary antibody (1:1000 dilution using PBS).
8. Wash the cells three times with 3 mL PBS and then remove the PBS using suction.
9. Mount the coverslip on the slide glass with mounting medium.
10. Capture cell fluorescence consecutively for E-Cadherin and N-Cadherin (emission range: 500–550 nm), and DAPI (emission range: 425–475 nm) using the laser scanning confocal microscope.

### 3.7. Statistical Analysis

**Figure 1:**
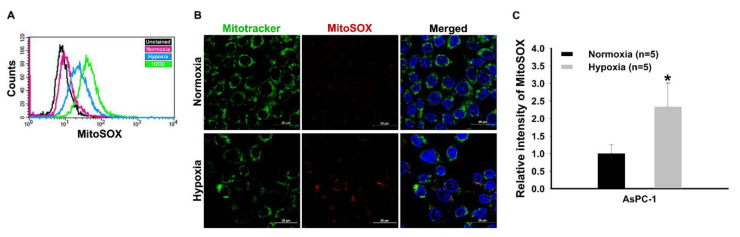
Flow cytometry and fluorescence-activated cell sorting analysis and immunofluorescence images for the detection of reactive oxygen species (ROS) in pancreatic cancer cells. (**A**) Mitochondrial superoxide indicator (MitoSOX)-based flow cytometry and fluorescence-activated cell sorting analysis detecting mitochondrial ROS. H_2_O_2_ was used for positive control. (**B**) Immunofluorescence staining for detecting mitochondria reactive oxygen species (ROS) (MitoSOX). Mitochondria tracker (Mitotracker) and MitoSOX fluorogenic dye accumulation in pancreatic cancer cells under hypoxia. Each pixel exposure time 0.1 s. Scale bar = 25 μm. (**C**) Quantitative analysis for mean fluorescent intensity of ROS generation between normoxia and hypoxia. The results were performed by quintuplicate obtaining repetitive results. A total of five cells with relatively high fluorescence intensity on one slide of each group was considered as a single analysis. * *P* < 0.05 vs. normoxia. Results are mean ± SD.

**Figure 2:**
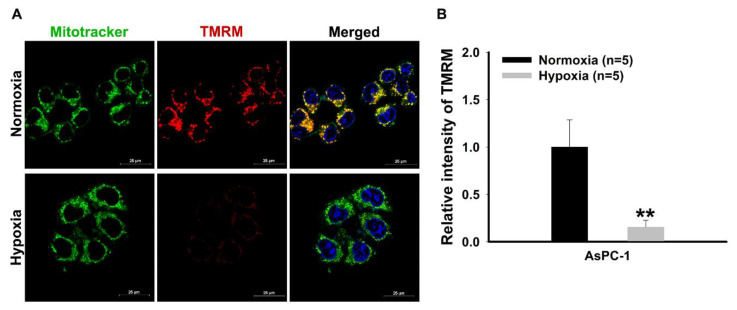
Representative immunofluorescence images for the detection of active mitochondria in pancreatic cancer cells. (**A**) Immunofluorescence staining detection of active mitochondria. Mitochondria tracker (mitotracker) and tetramethylrhodamine (TMRM) fluorogenic dye-stained accumulation with intact membrane potential in pancreatic cancer cells. Each pixel exposure time 0.1 s. Scale bar = 25 μm. (**B**) Quantitative analysis for mean fluorescence intensity of TMRM red fluorescence comparing between normoxia and hypoxia. The results were performed by quintuplicate obtaining repetitive results. A total of five cells with relatively high fluorescence intensity on one slide of each group was considered as a single analysis. ** *P* < 0.001 vs. normoxia. Results are mean ± SD.

**Figure 3:**
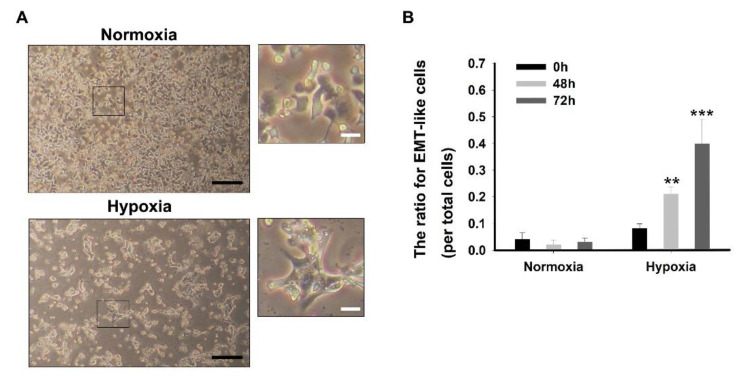
Representative morphological change by the epithelial–mesenchymal transition of pancreatic cancer cells. (**A**) Representative optical microscope pictures of the pancreatic cancer cells under hypoxia. Black scale bar = 50 μm; White scale bar = 10 μm. (**B**) Quantitative analysis for the ratio of epithelial to mesenchymal transition like cells per total cells. The results were performed by quintuplicate obtaining repetitive results. ** *P* < 0.001 vs. all normoxia and 0 h hypoxia; *** *P* < 0.001 vs. all normoxia, 0 h hypoxia, and 48 h hypoxia. Results are mean ± SD.

**Figure 4:**
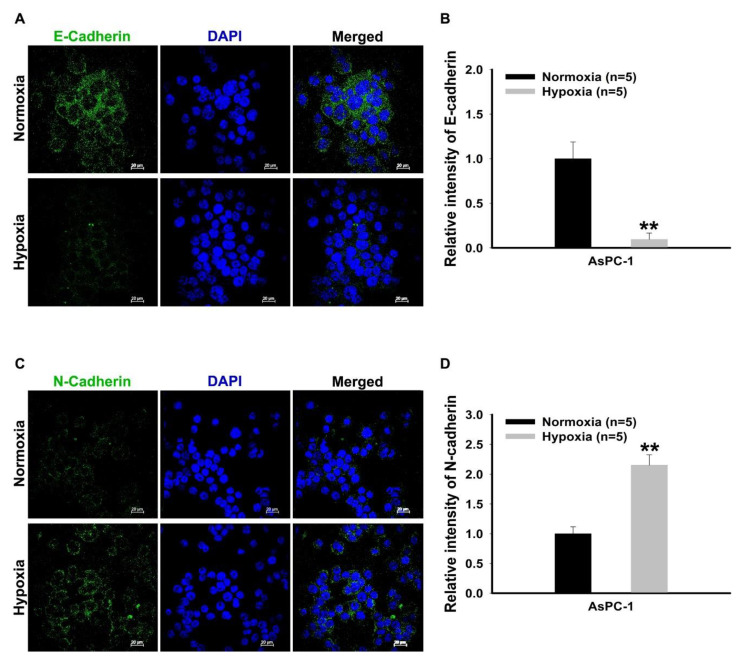
Representative immunofluorescence images for the detection of E-Cadherin and N-Cadherin in pancreatic cancer cells. (**A**,**C**) Immunofluorescence staining detection of E-Cadherin and N-Cadherin. Each pixel exposure time 0.1 s. Scale bar = 25 μm (**B**,**D**). Quantitative analysis for mean fluorescence intensity of E-Cadherin and N-Cadherin comparing between normoxia and hypoxia. The results were performed by quintuplicate obtaining repetitive results. A total of five cells with relatively high fluorescence intensity on one slide of each group was considered as a single analysis. ** *P* < 0.001 vs. normoxia. Results are mean ± SD.

Data were presented as mean ± standard deviation. The student’s t-test was used to calculate statistical significance between the groups in Figures 1C, 2B and 4B,D. One-way ANOVA was used to calculate statistical significance between the groups in Figure 3B.

## 4. Expected Results

Figure 1 shows the results of flow cytometry and fluorescence-activated cell sorting and immunofluorescence regarding the expression of ROS following the culture of pancreatic cancer cells in a normoxia or hypoxia condition for 72 h. The results of mitochondrial superoxide indicator-based flow cytometry and fluorescence-activated cell sorting showed that mitochondria ROS in pancreatic cancer cells tended to increase slightly in normoxia as compared to the unstained group (Figure 1A; pink line). Mitochondria ROS was further increased in hypoxia as compared to the unstained and normoxia groups (Figure 1A; blue line). The endogenous ROS hydrogen peroxide (H_2_O_2_) was used as a positive control (Figure 1A; green line). For the immunofluorescence analysis, intracellular mitochondria were stained with mitochondria tracker (Mitotracker; green) and mitochondrial superoxide indicator (MitoSOX; red) (Figure 1B). The mitotracker image shows that mitochondria in pancreatic cancer cells were found normally regardless of normoxia or hypoxia (Figure 1B). Meanwhile, it was observed that the staining level of the mitochondria ROS was increased under hypoxia, and the merged photo shows the colocalization of mitochondria tracker and mitochondrial ROS in the pancreatic cancer cells (Figure 1B). The quantitation of the fluorescent intensity of MitoSOX revealed a significant increase in mitochondria ROS under hypoxia (Figure 1C).

In Figure 2, the results regarding the detection of mitochondria function with active membrane potential are shown. Mitochondrial membrane potential was investigated with TMRM, which is a cell-permeant dye that accumulates in active mitochondria with intact membrane potentials (Figure 2). It was observed that the staining level of TMRM was decreased under hypoxia, and a merged photo shows the colocalization of mitochondria tracker and TMRM in the pancreatic cancer cells (Figure 3A). Little colocalization of TMRM was observed in the hypoxia condition. The quantitation of the intensity of TMRM staining revealed a significant decrease in mitochondrial function under hypoxia (Figure 2B).

Figure 3 shows that the morphological change of the pancreatic cancer cells under hypoxia. The results indicated the exhibiting a mesenchymal phenotype with the disappearance of defined cell-cell contacts, growing individually, and spindle-like morphology [10,11]. The phenotypes associated with EMT could be initially detected by direct microscopic observations of the monolayer cultures [10] (Figure 3A). The ratio of EMT-like cells per total cells were significantly increased to 0.21 ± 0.027% and 0.39 ± 0.092% after 48 h and 72 h under hypoxia as compared to the 0 h (0.08 ± 0.027) hypoxia and all normoxia groups, respectively (Figure 3B).

In Figure 4, the results regarding the fluorescent detection of E-Cadherin and N-Cadherin are shown. The fluorescent expression of E-Cadherin and N-Cadherin is shown in Figure 4A, it was observed that the fluorescence level of E-Cadherin was decreased while the level of N-cadherin was increased, under hypoxia (Figure 4A,C). The quantitation of the intensity of E-Cadherin and N-Cadherin revealed a significant decrease of a mesenchymal-epithelial transition (MET) and a significant increase of an EMT, under hypoxia (Figure 4B,D).

Two of the most important points of this protocol are ensuring the proper preparation of cancer cells and maintaining a hypoxic environment. Since human-derived pancreatic cancer cells are highly metastatic, they have very strong mesenchymal characteristics, and it can induce EMT through the hypoxic environment without special anthropogenic control. The growth of cancer cells is very rapid in the optimal culture condition with sufficient O_2_ consumption, such as normoxia. However, under hypoxia, even if the components of the media are adjusted to be equal to normoxia, the rate of death is relatively high. Therefore, it is necessary to appropriately control the number of pancreatic cancer cells when cultured in each condition. The purpose of this study was not to observe the viability between normoxia and hypoxia in the same number of cells. Therefore, in order to increase the yield of viable cells under hypoxia for various potential analyses at the end of the study, two to three times more cells than in the normoxia should be cultivated. Mitochondrial superoxide indicator-based assay is widely used to detect ROS in mitochondria specifically. Therefore, it is necessary to use hydrogen peroxide (H_2_O_2_) as a positive control for ROS generation in mitochondria to distinguish whether or not the mitochondrial superoxide indicator is well introduced. In this study, we used mitotracker green as a method to track the intracellular mitochondrial. Therefore, the increase in mitochondrial-specific ROS can be confirmed by double staining (Yellow) of mitochondrial superoxide indicator and mitotracker green (Figure 1B and Figure 2A). Active mitochondrial membranes maintain a difference in electrical potential between the interior and exterior of the organelle, which is referred to as a membrane potential [12]. TMRM is a cell-permeant, cationic, fluorescent dye that is readily accumulated by active mitochondria. The accumulation of TMRM was decreased by mitochondrial dysfunction with losing membrane potential under hypoxia. Since ROS generation occurs very rapidly under hypoxia, it does not dependent on the time flow. However, mitochondrial dysfunction with losing membrane potential occurs gradually after incubation under hypoxia. While this study has to be cultivated continuously under hypoxia, it is very difficult for cancer cells to grow normally under long-term hypoxia. Even if morphological changes could be observed more than four days, the death rate of the cancer cells will increase rapidly, making observation will be almost impossible. The cytomorphological results show that the number of EMT-like cells rapidly increases after 72 h (three days) under hypoxia. As shown in Figure 3A, there is a clear difference in the cytomorphology under hypoxia as opposed to the densely growing cells in normoxia; specifically, the cancer cells are growing sporadically under hypoxia. This phenomenon is not only a process in which EMT-induced epithelial cells lose cell polarity and intercellular adhesion, but also a process in which the total number of cells is decreased due to cell death. When pancreatic cancer cells are cultured under hypoxia, it can allow for a more accurate study of the characteristics of malignancy due to the EMT induction. Further, the accuracy of results was distinguished by the investigation of the expression of E-Cadherin and N-Cadherin in pancreatic cancer cells depending on the culture condition between normoxia and hypoxia. Especially in cancer cells with a strong EMT feature, the expression of N-Cadherin tends to be increased [13]. 

According to our results, up to 72 h under hypoxia was sufficient to confirm the mesenchymal feature of pancreatic cancer cells without intricacy control for EMT induction. Therefore, applying this protocol, tracking the changes in mitochondria membrane potential and ROS generation would be a potential supporter for deriving the result of EMT analysis besides examining the expression of previously known markers in a lab where confocal or FACS can be utilized.
